# Temporary Ureter Occlusion with Simultaneous Urinary Diversion via a Single-Access Route Using a 4-French Balloon Catheter and a Pigtail Nephrostomy Drainage Catheter

**DOI:** 10.3390/medicina60060975

**Published:** 2024-06-13

**Authors:** Chang Hoon Oh, Soo Buem Cho, Sang Lim Choi, Sungwon Kim, Hyeyoung Kwon

**Affiliations:** 1 Department of Radiology, Ewha Womans Mokdong Hospital, College of Medicine, Ewha Womans University, Seoul 07985, Republic of Korea; 01352@ewha.ac.kr; 2 Department of Radiology, Ewha Womans University Seoul Hospital, College of Medicine, Ewha Womans University, Seoul 07804, Republic of Korea; 3 Department of Radiology, Chung-Ang University Gwangmyeong Hospital, Gwangmyeong 14353, Republic of Korea; sanglim84@cauhs.or.kr; 4 Department of Radiology, Research Institute of Radiological Science, Yongin Severance Hospital, Yonsei University College of Medicine, Yongin 03186, Republic of Korea; kswpig@yuhs.ac; 5 Department of Radiology, Chungnam University Hospital, School of Medicine, Chungnam University, Daejeon 35015, Republic of Korea; hyeyoungkwon@cnuh.ac.kr

**Keywords:** ureter occlusion, urinary diversion, balloon catheter, nephrostomy, fistula

## Abstract

*Background and Objectives*: This study evaluated the efficacy and safety of temporary ureteral occlusion combined with urinary diversion using a single-access route created by inserting a balloon catheter through a pigtail nephrostomy drainage catheter. With this approach, we aimed to offer an alternative for patients with ureteral leaks who are suboptimal surgical candidates. *Materials and Methods*: This retrospective study included nine patients (eight of which were bilateral cases and one was unilateral, totaling seventeen cases) who underwent the surgery between September 2023 and March 2024. The method involved gaining percutaneous access to the pelvicalyceal system, inserting a 4-French Fogarty balloon catheter through a pigtail nephrostomy catheter, and inflating the balloon at the proximal or mid-ureter. *Results*: All 17 cases achieved technical successful with no major complications. The procedure effectively relieved symptoms associated with urinary leakage in most patients. However, the significant deflation of the balloon catheter occurred in five cases (29.4%), with three (17.6%) experiencing complete deflation. In these five cases, the final balloon size was 5.81 mm (range: 0–8.9 mm), confirming a 25.0% decrease in size from pre- to post-procedure. Ureteral occlusion was 28.3 d long on average (range: 8–57 d). All patients experienced symptom relief during temporary ureteral occlusion. Except for two patients lost to follow-up, three patients showed symptom improvement with only PCN and four patients underwent surgical closure of the fistula tract before or after balloon catheter removal. *Conclusions*: This study confirms that this approach is safe and effective.

## 1. Introduction

Urinary diversion facilitates the management of specific urological concerns, including vesical fistulas, severe dysuria due to bladder tumor irradiation or chemotherapeutic treatment, and ureteral leakage when a stent cannot be placed in the extravasation area [1]. The conservative or surgical management of a fistula in the lower urinary tract may be ineffective in up to 35% of patients because many are suboptimal surgical candidates. For such patients, percutaneous management is highly desirable as this procedure is associated with substantially fewer morbidities, faster recovery times, and lower complication rates [2,3,4].

Blocking urinary flow can be achieved percutaneously through several previously described techniques using coils and gelatin sponge particles (GSPs), N-butyl cyanoacrylate (NBCA), detachable balloons, nylon plugs, and bare and covered stents [2,3,4,5,6,7,8,9,10,11,12]. However, this approach is unsuitable for achieving reversible temporary ureteral occlusion and providing permanent embolic effects. Consequently, a different approach is required in order to provide temporary ureteral occlusion.

Park et al. achieved luminal dilatation for benign biliary strictures by inserting a balloon catheter into a drainage catheter, thus maintaining a single-profile catheter in the subcutaneous tract while using a balloon catheter internally [13]. Herein, we applied this technique and evaluated its ability to achieve simultaneous temporary ureteral occlusion with urinary diversion. We accomplished this using a balloon catheter indwelling at the ureter through the vascular sheath, hence requiring smaller skin and subcutaneous dilatation than previously described methods.

## 2. Materials and Methods

### 2.1. Patients

This retrospective single-center study was approved by the Institutional Review Board of Ewha Womans University (IRB no. EUMC 2023-10-046-002), which waived the requirement for patient informed consent. Nine patients (eight of which were bilateral cases and one was unilateral, totaling seventeen cases) with percutaneous temporary ureteral occlusion treated in our hospital between September 2023 and March 2024 were enrolled in this study. The inclusion criteria were as follows: persistent urine leakage, including leakage at the uretero-ileal anastomosis site after neobladder creation; vesico-vaginal or vesico-colic fistulas; urostomy site infection despite percutaneous nephrostomy (PCN) or repeated tube changes; and problems such as bladder or neobladder dysfunctions that were unresolved by the PCN tube or repeated tube changes. The following exclusion criteria were applied: an expected patient life expectancy of <3 months; prior kidney transplantation; and poor general health status (Eastern Cooperative Oncology Group performance status: grade 4).

### 2.2. Temporary Balloon Catheter Indwelling

For balloon catheter indwelling, percutaneous access to the pelvicalyceal system was punctured in Brodel’s nonvascular area under ultrasound and fluoroscopic guidance to reduce accidental arterial and venous renal complications. The major calyx was punctured with a 21 G Chiba needle, and 1 mL of contrast medium was slowly injected to perform pyelography and confirm the correct puncture. After inserting a 0.018-inch hair-wire (Slim wire, A&A M.D., Seongnam, Gyeonggi-do, Korea) into the Chiba needle, a 5-French (Fr) catheter was inserted and a pyelonephrogram was subsequently performed to evaluate the patient’s anatomy and ureter size. A 0.035-inch guidewire was inserted through the 5 Fr catheter and the 8.5 Fr pigtail PCN catheter was inserted subsequently (Cook Medical Inc., Bloomington, IN, USA).

**Figure 1:**
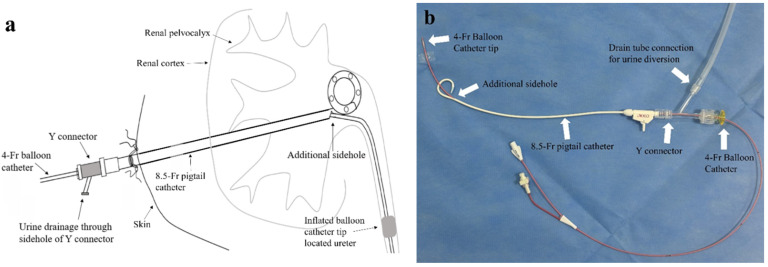
Images (**a**,**b**) show 4-French (Fr) balloon catheter indwelling using the 8.5 Fr pigtail percutaneous nephrostomy (PCN) catheter. A PCN catheter with an additional side-hole was inserted over the 0.035-inch guidewire in the renal pelvis. Then, a balloon catheter was inserted and passed through the side-hole of the PCN catheter with subsequent ballon inflation to reach the desired diameter at the proximal and mid-ureter.

We determined that the PCN catheter side-hole was too small; thus, an additional side-hole was created. Then, a 0.025-inch guidewire was passed through the new side-hole and a 4 Fr Fogarty balloon catheter (Edwards Lifesciences SA, Nyon, Switzerland) was inserted along the guidewire. The 4 Fr Fogarty balloon catheter tip was located at the proximal or mid-ureter for ureter occlusion. Typically, during balloon dilatation, the fluid injected into the inflation port is diluted at a 1:3 ratio with contrast medium and normal saline. This dilution is necessary because injecting an undiluted contrast agent into the inflation port can cause it to harden, making deflation of the balloon catheter impossible.Balloon dilatation did not exceed 9 mm to prevent ureteral rupture or ureteric stricture after balloon catheter removal. Subsequently, the balloon catheter inflation port was locked, and plaster was used to wrap the flow switch around the inflation port to minimize deflation. A Y-connector (OKAY II; NIPRO, Osaka, Japan) was used to hold the balloon and drainage catheter together during the indwelling period, and the Y connector valve was tightened. Then, the drain catheter was sutured and fixed to the skin (Figure 1). The inner diameter of the 8.5 Fr pigtail catheter was 1.85 mm and the outer diameter of the 4 Fr balloon catheter was 1.33 mm. Consequently, the cross-sectional area of the inserted pigtail catheter was approximately 1.93 times larger than that of the balloon catheter.

### 2.3. Follow-Up

All patients were clinically evaluated post-procedure using renal biochemical tests (e.g., blood urea nitrogen [BUN] or serum creatinine [Cr]) and plain abdominal radiography two times per week. Patients with unexpected symptoms, such as frequent urination, flank pain, dysuria, hematuria, or fever, were urgently evaluated. Pre- and post-balloon catheter removal, an antegrade ureterogram was obtained through the pigtail catheter to confirm persistent ureteral occlusion and the presence of ureteral stricture.

### 2.4. Study Endpoints

Technical success was defined as the effective placement of an inflated balloon catheter in the ureter with urine drainage through the vascular sheath, without contrast medium flowing through the ureter on final tubography. Blood clot retention was evaluated post-procedure by grading blood clots located in the renal pelvis on a three-point scale: grade 1: the retention of minimal or no blood clots in one or more calyces or infundibulum alone; grade 2: the retention of blood clots in less than half of the renal pelvis; and grade 3: the retention of blood clots in most of the renal pelvis and/or ureter. The significant deflation of the balloon catheter was defined as a >10% decrease in the balloon size when compared to that observed in the abdominal X-ray immediately post-procedure. Complications were classified as minor or major according to the Society of Interventional Radiology guidelines [14].

## 3. Results

Table 1 summarizes the clinical characteristics, technical success rates, fluoroscopy times, balloon location and duration of maintenance, blood clot retention grades post-procedure, and follow-up results of patients. Of the nine patients, eight and one underwent bilateral and unilateral procedures, respectively, totaling seventeen cases. Five patients had bladder cancer, three had cervical cancer, and one had metastatic ovarian cancer. There were several reasons why temporary ureteral occlusion needed: four patients had urinary system fistulas (including two vesico-colic and two vesico-vaginal fistulas, respectively), one had urethra anastomosis site leakage after pelvic exenteration and radical cystectomy with neobladder creation, one had ileostomy site infection, one had persistent intractable urgent urinary incontinence despite PCN placement, one had tumor recurrence at the neobladder with dysfunction, and one had bladder dysfunction due to bladder cancer with suspected perforation.

**Figure 2:**
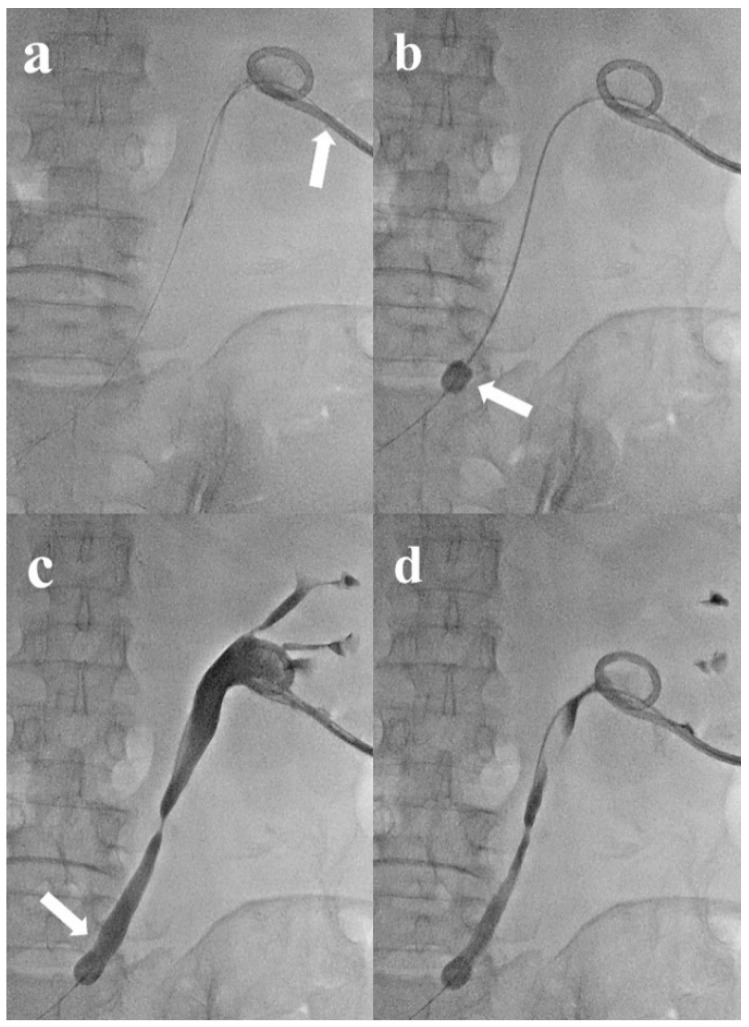
A 73-year-old woman with bladder cancer was referred for temporary ureteral occlusion at both ureters due to tumor recurrence at the neobladder with dysfunction. Image (**a**) shows an 8.5-French (Fr) pigtail nephrostomy catheter with an additional side-hole inserted via the lower pole of the left kidney and a 0.025-inch guidewire inserted through the additional side-hole (arrow). Image (**b**) shows 4 Fr balloon catheter subsequently inserted through the guidewire, and the inflated balloon catheter tip positioned at the proximal ureter (arrow). Image (**c**) illustrates that the tubography performed after balloon catheter inflation showed no contrast media flow to the distal portion past the balloon catheter (arrow), indicating a well-occluded ureter. Image (**d**) shows the evaluation of urine drainage via PCN catheter declamping and the subsequent evaluation of contrast media drainage without disturbance.

All 17 cases of temporary ureteral occlusion achieved technical success. Additionally, there was no occurrence of flank pain or worsening of hydronephrosis following insertion, and urine diversion through the pigtail catheter was successfully achieved in all cases. Hydronephrosis was graded as level 1 in 15 patients (88.2%) and level 2 in 2 patients (11.8%). The balloon catheter tip was positioned in the proximal and mid-ureter in 13 (76.5%) and 4 cases (23.5%), respectively (Figure 2). The initial balloon inflation size was 7.75 mm (range: 6.0–8.9 mm). BUN levels decreased by 28.8% from an average of 27.1 pre-procedure to 19.3 post-procedure. Cr levels also decreased by 17.9%, from 1.23 to 1.01. No patients developed major complications. However, the significant deflation of the balloon catheter occurred in five cases (29.4%), with three cases (17.6%) experiencing complete deflation. In those cases, the final balloon size was mean 5.81 mm (range: 0–8.9 mm), revealing a 25.0% decrease between pre- and post-procedure sizes. No patient experienced any retraction of the PCN catheter post-procedure. Ureteral occlusion averaged 28.3 d (range: 8–57 days) and one patient (accounting for two cases) was lost to follow-up. All patients experienced symptom relief during temporary ureteral occlusion. Except for the two patients lost to follow-up, three patients showed symptom improvement. Four patients underwent surgical closure of the fistula tract before or after balloon catheter removal and maintained only PCN, which also resulted in symptoms improvement. Excluding the patient lost to follow-up, all others showed improved symptoms following temporary ureter occlusion or after surgical repair, allowing balloon catheter removal.

## 4. Discussion

The kidney is a highly vascular organ that may be injured during percutaneous interventions, potentially resulting in clinically significant hemorrhage. However, this is uncommon, with a reported hemorrhage rate requiring a transfusion of 1–4% and a < 1% rate of vascular injury, necessitating arterial intervention or nephrectomy [15]. Consequently, we adopted a single-access route by inserting a balloon catheter into a pigtail PCN catheter through an additional side-hole, reducing iatrogenic injury to the kidney while providing effective urinary diversion. In our study, technical success was achieved in all patients without encountering significant difficulties. The dual-catheter placement technique has been employed to treat benign biliary strictures, allowing the stricture site to be dilated to a wider diameter than that achieved by previous methods maintaining a single large-profile catheter. However, it still necessitates large-diameter subcutaneous tract dilatation [16]. To address this issue, another study introduced a balloon catheter into a drainage catheter, achieving a smaller profile for the skin and subcutaneous tissue [13]. With this technique, the inflation size at the target stricture site is determined by the balloon diameter. Moreover, the catheter has a small profile through skin and subcutaneous tissue, making it sufficient for the balloon catheter to pass. In our study, a 4 Fr balloon catheter was inserted into an 8.5 Fr PCN catheter to effectively accomplish urine diversion and simultaneous ureter occlusion. Pre- and post-procedure BUN and Cr decreased by 28.8 and 17.9%, respectively, and the absence of flank pain or signs of hydronephrosis. Furthermore, during the period of temporary ureteral occlusion using the balloon catheter, all patients experienced symptom relief. This indicated that urine drainage was effective, while ureteral occlusion was also successful.

Previously, because of persistent urinary leakage or surgical contraindications in most patients, conservative treatment with ureteral occlusion has been attempted [17]. Generally, PCN is the preferred method for urine diversion in cases of urine leakage either due to traumatic iatrogenic ureteral injury, the presence of inflammatory, or malignant urinary fistulas (most commonly including vesicovaginal and ureterovaginal); however, ureterocutaneous and ureteroenteric fistulas can also occur [18,19]. When continued leakage persists, PCN placement may be necessary as it is more capable of diverting urine from the injured area. However, nephrostomy alone does not completely cease urine drainage into the urinary bladder. Ureteral occlusion techniques have varied success in preventing antegrade urine flow [20]. In patients with advanced inoperable gynecologic or pelvic tumors, or for other reasons where urine continues to flow through the ureter, the recurrence of symptoms and extended treatment periods may necessitate surgical repair or ureter occlusion. In cases when surgery is contraindicated, the patient refuses surgery, or the patient has a limited life expectancy, percutaneous ureter occlusion has been explored in various studies. Techniques such as coils, GSP, NBCA, and vascular plugs have been used to achieve occlusion [2,3,4,5,6,7,8,9,10,11,12,21,22]. Most methods involve the embolization of the ureter proximal to the site of urine leakage or fistula using a permanent embolic agent. Some studies have also targeted the fistula tract directly, employing a vascular plug for embolization [22]. However, a major drawback is the permanent nature of the ureteral occlusion and lifelong necessity for PCN catheter placement [23].

In a study by Horenblas et al., a Foley balloon catheter was used to perform temporary ureter occlusion [17]. The Foley catheter features a thin latex balloon, which inflates into a slightly flattened sphere in the ureter, ensuring complete occlusion with the minimal compression of the urothelium. In contrast, angioplasty catheters have a more elongated and rigid balloon, which might not occlude as effectively and pose a greater risk of ureteric wall pressure necrosis. Effective ureteral occlusion generally requires adequate urinary diversion, typically through a PCN. Without it, the ureter may dilate, causing the balloon catheter to lose contact or migrate [24]. Angioplasty balloons are generally less effective than the more flexible latex Foley catheters used for occlusion. We used a Fogarty balloon catheter equipped with a low-profile compliance balloon to perform the procedure. The Fogarty balloon catheter has a non-compliant low-pressure balloon that assumes a spherical shape when inflated. Although the characteristics of this compliance balloon are similar to those of a Foley catheter, its low profile offers the advantage of easy insertion through a single-access route inside a pigtail PCN catheter. A disadvantage of balloon catheters is the necessity of frequent revisions due to recurrent urinary leakage [17]. In our study, the significant deflation of the balloon catheter occurred in five cases (29.4%), with three cases (17.6%) experiencing complete deflation. A previous study suggested that the reasons for spontaneous balloon deflation include catheter breakage with a subsequent loss of insufflation fluid or the dilation of the ureter proximal to the balloon due to inadequate drainage through the PCN catheter [17]. Further studies are needed to ensure that, even with long-term placement, deflation does not readily occur.

Günther et al. [24] examined dog ureters after 3 weeks of occlusion. Histology showed the flattening and mild inflammation of the urothelium. Although no reports have been published on ureteric wall necrosis after the long-term use of balloon occlusion, this could be expected on theoretical grounds. However, a previous study demonstrated that even after 5 months of ureteric occlusion with a balloon catheter, no evidence of ureteric wall necrosis was observed [17]. Additionally, while not observed in our study, other potential problems may occur, such as issues arising from PCN catheter dislodgement leading to balloon catheter retraction. Previous reports showed varied nephrostomy tube types and respective dislodgment rates ranging from 1 to 30% [25,26]. Although skin sutures are used to secure the catheter, the possibility of dislodgement still exists. It is crucial to instruct and educate patients to ensure that the catheter is not pulled, and continuous follow-up is necessary.

This study has its limitations. First, it was retrospective in nature, which prevented control over certain conditions, such as the day of temporary ureter occlusion, as well as the time between balloon catheter removal and surgical closure. Second, the heterogeneous patient group and the number of included patients was small. Therefore, defining clinical success in this study was ambiguous. We focused on whether symptoms were relieved during the occlusion period and on controlling symptoms that were previously unmanageable with only PCN. Due to these limitations, comparisons with a control group and investigation of risk factors were not feasible. Consequently, additional studies with more cases are warranted to further assess the feasibility of this method. Finally, the short follow-up period and the duration of balloon catheter maintenance (mean: 28.3 d) present challenges for assessing the potential complications or effectiveness of the balloon catheter.

## 5. Conclusions

In conclusion, we found that using a non-compliance balloon catheter and PCN drainage catheter for temporary ureter occlusion with simultaneous urinary diversion via a single-access route was safe and effective.

## Figures and Tables

**Table 1:** Patients’ clinical characteristics, technical success rates, fluoroscopic times, maintenance of balloon location and duration, grades of blood clot retention after the procedure, and follow-up results.

No.	1	2	3	4	5	6	7	8	9
Age/Sex	F/44	M/77	F/42	F/76	F/80	M/63	F/59	M/87	F/73
Underlying disease	Cervical cancer	Bladder cancer	Cervical cancer	Cervical cancer	Bladder cancer	Bladder cancer	Ovarian cancer with metastasis	Bladder cancer	Bladder cancer
Symptoms and Reason for Occlusion	Urine leaking through the vagina due to vesicovaginal fistula	Repeatedly persistent ileostomy site infection despite PCN placement	Urethra anastomosis site leakage after pelvic exenteration and radical cystectomy + neobladder creation	Feces drainage from foley catheter due to vesicocolic fistula after radiation therapy	Persistent intractable urgent urinary incontinence despite PCN placement	Urine leakage from rectum due to vesicocolic fistula	Urine leaking through the vagina due to vesicovaginal fistula	Abdominal pain and discomfort due to bladder dysfunction with R/O microperforation despite PCN placement	Voiding discomfort due to tumor recurrence at neobladder
Direction	Bilateral	Bilateral	Bilateral	Bilateral	Unilateral (Right)	Bilateral	Bilateral	Bilateral	Bilateral
Hydronephro-sis grade	1/1	1/1	1/1	2/1	1	1/2	1/1	1/1	1/1
Blood clot retention grade	1/2	1/1	2/1	1/1	1	1/1	2/2	1/1	1/1
Balloon location	Proximal/Proximal	Proximal/Mid	Proximal/Proximal	Mid/Mid	Proximal	Proximal/Proximal	Proximal/Proximal	Proximal/Mid	Proximal/Proximal
Initial balloon size (mm)	7.3/7.1	8.6/8.5	7.5/7.8	8.3/7.1	8.9	8.1/7.6	7.3/6.0	6.7/7.7	8.9/8.4
Final balloon size (mm)	6.7/4.5	8.4/8.3	6.8/5.1	8.1/7.1	-	6.5/6.9	7.2/5.9	-/-	8.9/8.4
Deflation of balloon	N/N	N/N	N/N	N/N	Y	Y/N	N/Y	Y/Y	N/N
Technical success	Y/Y	Y/Y	Y/Y	Y/Y	Y	Y/Y	Y/Y	Y/Y	Y/Y
Urine drainage	Y	Y	Y	Y	Y	Y	Y	Y	Y
Complication	N	N	N	N	N	N	N	N	N
Duration (days)	17	35	23	18	57	38	46	8	13
Remarks	Balloon catheter removal due to no residual symptoms after POD 17 → No recurrence of significant symptoms, but residual defects were found on cystostoscopy → Surgical closure after 3 months	Dermatological improvement of the ileostomy site infection during ureter occlusion → Re-procedure was performed3 times due to the recurrence of symptoms → Balloon catheter removal and retention of only the PCN catheter without the recurrence of symptoms	No additional urine leakage from the urethral anastomosis site → Balloon catheter removal on POD 23 and surgical revision (neobladder → ileal conduit creation) after POD 26	Surgical repair of the urinary bladder on POD 4 → Balloon catheter removal on POD 18 without recurrent feces drainage from the foley catheter	Deflated balloon catheter was used on POD 15 → Re-procedure (temporary ureteral occlusion) → Improved symptoms → Balloon catheter removal and keeping only the PCN catheter → No recurrence of symptoms	Repeated procedure due to deflated balloon catheter (right) on PODs 8, 16, and 30 → Improved symptoms and patent balloon catheter on abdominal X-ray → Follow-up loss after POD 38	Repeated procedure due to drainage catheter retraction (right) and deflated balloon catheter (left) on POD 9 → Balloon catheter removal on POD 46 and keeping only the PCN catheter → Surgical closure after 1 months	Improved symptoms but deflated balloon catheter (both) on abdominal X-ray POD 8 → Balloon catheter removal and keeping the PCN catheter without the recurrence of symptoms	Improved symptoms during balloon catheter placement, but follow-up loss after POD 13

Abbreviation: PCN: percutaneous nephrostomy, Fr: French, POD: post-operative day.

## Data Availability

The data presented in this study are available on reasonable request from the corresponding author.
